# Eveningness in Energy Intake among Adolescents with Implication on Anthropometric Indicators of Nutritional Status: The CRO-PALS Longitudinal Study

**DOI:** 10.3390/nu12061710

**Published:** 2020-06-07

**Authors:** Josip Rešetar, Danijela Pfeifer, Marjeta Mišigoj-Duraković, Maroje Sorić, Jasenka Gajdoš Kljusurić, Zvonimir Šatalić

**Affiliations:** 1 Faculty of Food Technology and Biotechnology, University of Zagreb, Pierottijeva 6, 10000 Zagreb, Croatia; jresetar@pbf.hr (J.R.); dpfeifer@pbf.hr (D.P.); zsatalic@pbf.hr (Z.Š.); 2 Faculty of Kinesiology, University of Zagreb, Horvaćanski zavoj 15, 10000 Zagreb, Croatia; marjeta.misigoj-durakovic@kif.hr (M.M.-D.); maroje.soric@kif.hr (M.S.); 3 Faculty of Sport, University of Ljubljana, Gortanova ulica 22, 1000 Ljubljana, Slovenia

**Keywords:** eveningness, energy intake, timing, adolescents, obesity, anthropometry

## Abstract

Shifting of energy intake towards a later time in the day is associated with an increased risk of obesity in adults. However, there is a lack of data for adolescents. The aim of this study was to investigate adolescents eveningness in energy intake (EV) and its association with anthropometric indicators of nutritional status. This investigation was based on results from the Croatian physical activity in adolescence longitudinal study (CRO-PALS). The cohort included 607 adolescents (50.25% females and 49.75% males) who were assessed at the age of 15/16 and 18/19. A single multi-pass 24-h recall was used as a dietary assessment method, while anthropometric indicators of nutritional status included body mass index (BMI), waist to hip ratio (WHR) and the sum of four skinfolds. The School Health Action, Planning and Evaluation System (SHAPES) questionnaire was used to assess active daily energy expenditure and sedentary behaviors. EV was significantly higher at 18/19 years compared to 15/16 years in whole population (*p* < 0.01), among male adolescents (*p* < 0.01), but not among female adolescents (*p* > 0.05). Although a significant correlation between EV and WHR was found in females at the age of 15/16 (*p* < 0.01), the results of this study suggest that EV has no or a minor effect on anthropometric indicators of nutritional status in adolescence.

## 1. Introduction

Obesity is influenced by many determinants and risk factors—genetic variations, epigenetics, endocrine diseases, microbial dysbiosis, birth weight, diet, physical activity, sleep, energy expenditure, etc. [1]—and is one of the most challenging problems the public health is faced with at present. The rising prevalence of overweight and obesity in adolescence is also a concern [2], since it is well documented that overweight or obese adolescents are at increased risk of becoming overweight or obese adults [3]. Additionally, the number of adipocytes, which is the major determinant for fat mass in adults, seems to be set during childhood and adolescence and remains the same in adulthood [4]. Hence, in order to stop the obesity pandemic outbreak in adulthood, the focus should be placed on preventive interventions at a younger age.

Numerous scientific papers have unquestionably shown that energy intake surplus can lead to weight gain and, consequently, obesity [5]. However, it is quite unknown whether specific daytime energy intake/distribution plays a role in the risk of obesity occurrence. It can be noted that the time of eating differs among different countries and cultures, which complicates the understanding of timing–health-implications relationship [6,7]. According to previous studies, occasions like breakfast-skipping, late lunch, or late dinner are associated with a higher risk of developing obesity and metabolic diseases [8]. In a Swedish study by Berg et al., it was found that eating at the late hours was associated with an odds ratio (OR) for obesity of 1.62, with a 95% confidence interval (CI), compared with no eating at the late hours [9]. Late-night eating, accompanied by breakfast-omitting, was also associated with a higher risk of developing metabolic syndrome in Japanese adults [10]. Not surprisingly, it has been observed by US and German authors that higher evening energy intake was correlated with higher total daily energy intake [11,12].

One of the aspects of the broad general factor of timing is eveningness in energy intake (EV), which is a relative indicator for energy intake shifting, first introduced by Diederichs et al. [12]. The use of EV as a variable can implicate if energy distribution is shifted more to the first or to the last part of the day and, therefore, could be used as a potential risk predictor for obesity development. Even though the evidence suggests that, in recent years, energy intake has shifted towards later part of the day [13], which gives rise to a higher risk of cardiometabolic outcomes in adults, the effect of energy intake shifting in adolescence is still uncertain. A factor that should be taken into consideration when analyzing energy intake distribution in adolescents is a shift in adolescents’ chronotype with age—which can result in a shift in energy intake to the last part of the day [14]. To distinguish whether the shift in energy intake is primarily the result of physiological changes typical for adolescence and/or a risk factor for obesity onset, anthropometrical data should be included in analysis.

The aims of our study were to (i) investigate EV and (ii) examine the associations between EV, anthropometric indicators of nutritional status and physical activity in a cohort of secondary school students (assessed at the age of 15/16 and 18/19). We hypothesized that both female and male adolescents would express a shift in energy intake towards later hours of the day as they age, and that a potentially pronounced shift of energy intake among adolescents would be associated with deterioration of anthropometric indicators of nutritional status.

## 2. Materials and Methods

### 2.1. Subjects

This study is based on results from Croatian physical activity in adolescence longitudinal study (CRO-PALS). The CRO-PALS study is an observational study on a random sample of adolescents in the city of Zagreb (Croatia). For the purpose of the study, all 86 secondary schools in Zagreb were stratified according to a type of school (gymnasiums, vocational schools, or private schools). In the first stage of sampling, students were separated by means of two-stage stratified random selection, including stratification based on the proportion of different types of schools, as well as the average number of students who enrolled in the first grade. The first stage of sampling resulted in 14 randomly selected secondary schools in the city of Zagreb, 13 public (eight vocational and five grammar schools), and one private grammar school, with a total of 2827 students. During the second stage of sampling, in each of the selected schools, half of the 1st grade class units were randomly selected, resulting in 1408 students. All students in selected classes were invited to participate in the study, with no specific inclusion/exclusion criteria being applied. After participation forms were distributed among students and their parents, 903 (64%) adolescents agreed to take part in the study. Therefore, our sample involved almost 10% of all children enrolled in the first grade of secondary schools in the city of Zagreb. The study started in spring 2014, according to the Declaration of Helsinki, and all procedures were approved by the Ethics Committee of the Faculty of Kinesiology, University of Zagreb (No. 1009-2014).

The socio-economic status (SES) was assessed by parents’ perception of their socio-economic standing using categories ranging from 1–5 (1—Much lower than average; 2—Lower than average; 3—Average; 4—Higher than average; 5—Much higher than average). When participants enrolled in the final year of high school education (third or fourth, depending on the duration of each high school program), another examination was performed in spring 2016, i.e., spring 2017. The high schools investigated in this study dominantly conduct a four-year high school education program, 95.9%, respectively, in which case retest was performed in spring 2017. After the survey was carried out, part of the respondents had to be removed from further processing due to incomplete data. In this research, the data for 607 subjects (50.25% females and 49.75% males) who provided complete data both at the age of 15/16 and 18/19 were finally processed. The average age of the whole population at 15/16 and 18/19 age was 15.6 and 18.6 years, respectively (Table 1).

### 2.2. Assessment of Diet

As a dietary assessment method, a single multi-pass 24-h recall in the form of an interview was used [15]. This method was also used in similar studies with adolescents [16]. Food intake was collected solely for weekdays, excluding weekends and Fridays, because Friday can be considered as a transition day with a resemblance to days of the weekend. The interview was carried out by the graduate Nutrition students at the Faculty of Food Technology and Biotechnology, University of Zagreb. The interview was conducted through five standardized steps [15]:

(1) Generating a working list of consumed food;
(2) Updating the list that the respondent subsequently recalled;
(3) Specifying the time and circumstances of the meal;
(4) Asking for more detailed information on consumed food and beverages;
(5) Final verification of the collected data.

As a portion size estimation aid, a national food portion booklet containing food photography showing three serving sizes (small, medium, and large) was provided to respondents [17]. The chemical composition of consumed food was estimated using a combination of national, Danish and US nutritional tables [18,19,20]. The chemical composition of complex dishes was calculated on the basis of traditional recipes [21].

### 2.3. Definition of Eveningness in Energy Intake

Analysis of energy intake distribution during the day was based on dividing food consumption into morning, lunch, and evening intake. The definition of morning and evening energy intake, as well as eveningness in energy intake, was the same as described in the study by Diederichs et al. [12]. Morning intake was defined as any food and drinks consumed from evocation until 11 a.m., while evening intake was considered any food and beverages taken in after 6 p.m. until going to sleep. Lunch energy intake includes food and beverages eaten between 11.01 a.m. and 5.59 p.m. Eveningness in energy intake is calculated by subtracting morning from evening energy intake. These energy intake variables were calculated by following equations (Equations (1)–(5))

(1) $$TDEI=\sum E_{D}$$

(2) $$EI_{morning}=\sum E_{before\;11a.m.}$$

(3) $$EI_{lunch}=\sum E_{11.01a.m.-5.59p.m.}$$

(4) $$EI_{evening}=\sum E_{after\;6p.m.}$$

(5) $$EV=Eveningness=EI_{evening}-EI_{morning}$$

where:

*TDEI*—Total daily energy intake (kcal);

*E_D_*—The energy (kcal) from food and beverages consumed during the day;

*EI_morning_*—Morning energy intake (kcal);

*E_before_*_11*a.m.*_—The energy (kcal) from food and beverages consumed from evocation until 11 a.m.;

*EI_lunch_*—Lunch energy intake (kcal);

*E*_11.01*a.m.*–5.59*p.m.*_—The energy (kcal) from food and beverages consumed between 11.01 a.m. and 5.59 p.m.;

*EI_evening_*—Evening energy intake (kcal);

*E_after_*_6*p.m.*_—The energy from food and beverages consumed after 6 p.m. until bedtime.

### 2.4. Anthropometric Data

Participants’ body weight was measured while they were barefoot with their shorts and T-shirts on, with the standard scale set to the nearest 0.1 kg. Height was taken, using an anthropometer, to the nearest 0.1 cm (GPM, Siber-Hegner and Co., Zurich, Switzerland). Afterwards, body mass index (BMI) was calculated as body weight in kilograms divided by body height in meters squared (kgm^−2^). Age and gender-specific BMI percentile curves were used to distinguish between normal weight and overweight/obese adolescents (Table 2) [22].

Waist circumference was measured using a tape measure to the nearest 0.5 cm at the midpoint between the lowest rib and the iliac crest. Hip circumference was measured using a tape measure to the nearest 0.5 cm at the point of the greater trochanter. Waist to hip ratio (WHR) was calculated as waist circumference divided by hip circumference. Skinfold thickness measurements were taken on the right side of the body at the following sites to the nearest 0.2 mm using Harpenden skinfold caliper (British indicators, West Sussex, UK):

1. Triceps—At the back of the upper arm, halfway between the acromion process and the olecranon process;
2. Biceps—At the front of the upper arm; at the same level as the triceps;
3. Subscapular—About 2 cm below the lower angle of the scapula: a diagonal fold;
4. Suprailiac—At the iliac crest; in anterior axillary line plane.

All skinfold measures were taken in triplicate by a single, skilled lab technician. Median values of triplicate skinfold measures were used for analysis. The sum of four skinfolds (S4SF) was a variable chosen as an indicator of adiposity due to a more accurate reflection of adolescents’ adiposity compared to BMI [23].

### 2.5. Physical Activity

School Health Action, Planning and Evaluation System (SHAPES) questionnaire [24] was used to assess participants’ physical activity level, as well as sedentary behaviors. A module of the SHAPES questionnaire used in this research requests a 7-day recall of moderate physical activity (MPA) and vigorous physical activity (VPA). For each recalled day, participants indicated the number of hours (0–4 h) and 15-min increments (0–45 min) that MPA and VPA were performed. Average daily physical activity energy expenditure (AEE) was calculated according to equations provided by Wong et al. [24].

Sedentary behaviors were assessed through the 7-day recall of time spent: (1) playing computer/video games; (2) watching television/movies; (3) surfing the internet; (4) studying and doing homework; (5) listening to music; (6) reading; (7) playing instruments. As for physical activity, responses were provided by indicating the number of hours and 15-min increments that these seven activities were performed.

### 2.6. Statistical Analysis

Statistical analysis was performed using IBM SPSS Statistics software for Windows, Version 26.0 (IBM, Armonk, NY, USA) and RStudio open-source software for Windows, Version 1.2 (RStudio, Inc., Boston, MA, USA). Descriptive statistics were used to calculate the mean, median values and percentiles [25]. The two-tailed paired Student *t*-test was used to compare differences between the means of parametric continuous variables for the same subjects at the first and the last year of study. Statistical significance for categorical variables was calculated using the Pearson χ^2^ test. A significant difference was evaluated at a *p*-value of less than 0.05. The Spearman rank correlation coefficient was calculated between variables by using a two-tailed significance test for variables with the non-Gaussian distribution. The socio-economic status (SES), age, gender and total daily energy intake (TDEI) were used as covariates in the Generalized Estimating Equations (GEE) analysis.

Previously mentioned tools were used to establish correlations between observed parameters. In order to go forward and investigate what the expected change in an anthropometric parameter as a consequence of a certain EV or physical activity is, the classification and regression tree was applied. This tool helps to investigate the patterns in the large data, which presents the data mining approach, set to use the Chi-square Automatic Interaction Detection (CHAID) method with a significance level of 5% with the minimum parent size set as 2.

## 3. Results

The study sample consisted of 607 participants, 50.25% females and 49.75% males, which is the gender ratio in the general population [26]. The whole study sample was followed from the age of 15/16 to the age of 18/19. Energy intake data for the whole population and by age and gender groups are shown in Table 3.

**Figure 1:**
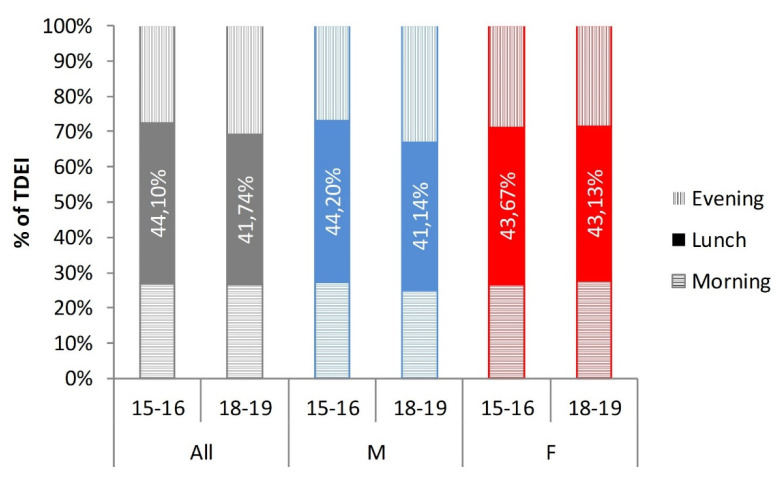
Morning, lunch, and evening energy intake contribution to total daily energy intake (TDEI) shown as a percentage of TDEI depending on age and gender of adolescents. The whole population is marked in grey, male population in blue and female population in red color.

Median total daily energy intake (TDEI) for the whole population was similar on the first and the last year of secondary school: 1908 kcal at 15/16 years and 1919 kcal at 18/19 years. Male participants had a higher median TDEI than females both at the age of 15/16 and 18/19 (Table 3). A statistically significant difference in TDEI between the first and last year of secondary school for females, as well as males, was not observed (*p* > 0.05). Observing the energy intake distribution during the day, it has been found that lunch energy intake (from 11.01 a.m. until 5.59 p.m.; *EI_lunch_*) contributes the most to TDEI (Figure 1). In male participants, at the age of 15/16, the highest contribution of *EI_lunch_* to TDEI has been noted (44.2%). A similar percentage of TDEI for *EI_lunch_* was observed for the whole population at the age of 15/16. The contribution of *EI_lunch_* to TDEI decreased from the age 15/16 to 18/19 in the whole population and in the male population of adolescents, while *EI_lunch_* contribution to TDEI in females was similar in both age. As for lunch energy intake, morning (*EI_morning_*) and evening energy intake (*EI_evening_*) in the female population did not differ much depending on participants age, whereas an increase in *EI_evening_* was noted for males and the whole population at the age of 18/19 (Figure 1).

**Figure 2:**
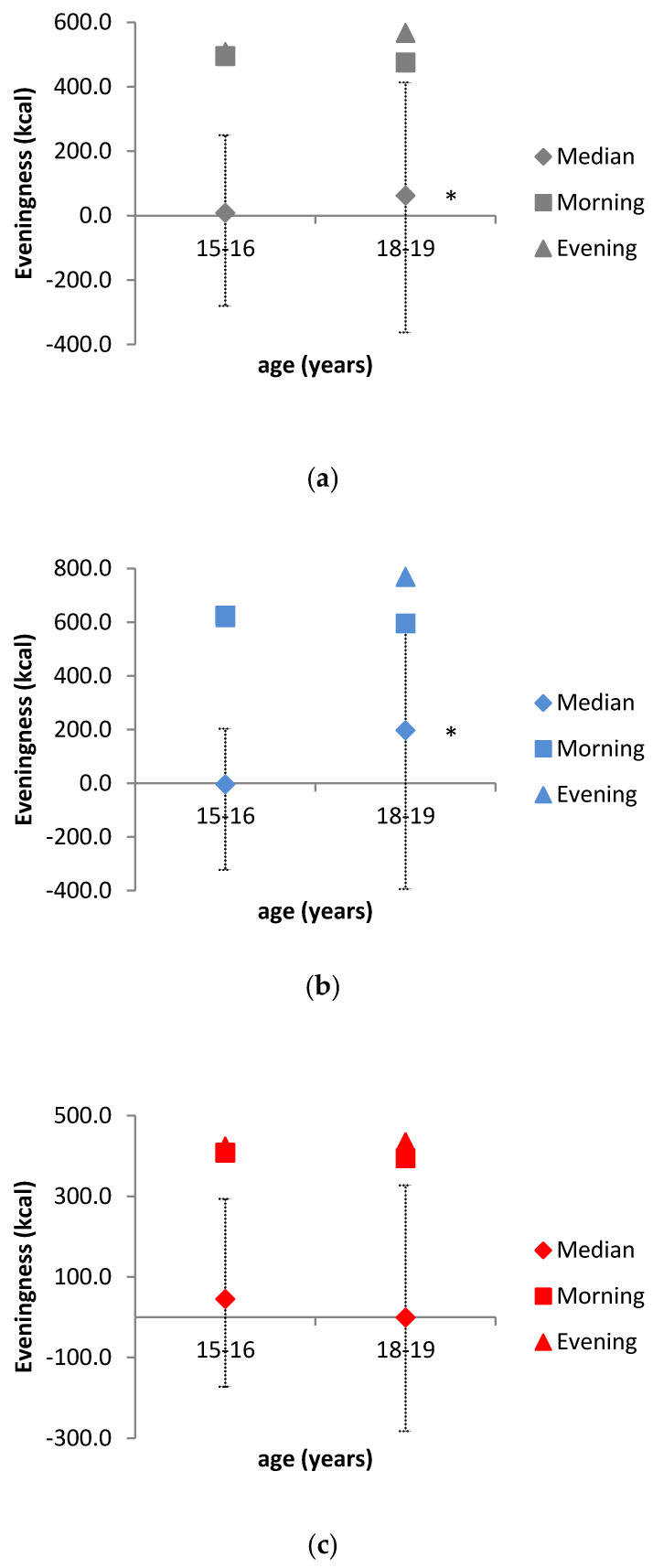
Eveningness in energy intake presented as the difference between evening and morning energy intake. ♦ Shows 50th percentile, while dotted lines represent range from 25th to 75th percentile. ■ indicates morning energy intake and ▲ represents evening energy intake. * represents a statistically significant difference between the age of 15/16 and 18/19 (*p* < 0.05): (**a**) whole population; (**b**) male population; (**c**) female population.

The highest difference between median morning and evening energy intake was observed in female participants at the age of 15/16, while at the age of 18/19 male subjects have shown a higher tendency to larger *EI_evening_*. More pronounced *EI_evening_* in 18/19 years old males is also seen in the increased EV (Table 3). As well as the median value of *EI_evening_*, the median value of EV increased, but the median value of *EI_morning_* decreased in the whole population from the age of 15/16 to 18/19 (Figure 2, Table 3). Moreover, in whole population, EV was significantly higher in 18/19-years compared to 15/16-years (*p* < 0.01). This trend was also observed in the male population of adolescents (*p* < 0.01), while the same was not found in the female population (*p* > 0.05) (Figure 2).

Changes in body mass index (BMI) in whole population show significantly higher values when adolescents are older (18/19 years) (*p* < 0.01), but changes differ between genders. The trend of higher BMI at the age of 18/19 was noted in male adolescents (*p* < 0.01), whereas the same trend was not found in females (*p* > 0.05). Regarding the difference in nutritional status in males, the percentage of overweight participants increased with age, but the percentage of obese male participants decreased. On the contrary, the percentage of overweight female participants was slightly lower at the age of 18/19, while the percentage of obese females increased (Table 4). A difference in the waist to hip ratio (WHR), in both female and male population, during the study, was not observed (*p* > 0.05). However, it is noted that male participants at the last year of secondary school had a slightly higher sum of four skinfolds (S4SF) than in the first year of their secondary school education. In the female population, S4SF slightly decreased with age, showing the opposite trend from the male population (Table 4). Still, no significant difference considering change in S4SF in both genders was found (*p* > 0.05). Furthermore, the difference between the first and the last year of adolescents’ secondary school education was observed for active energy expenditure during school days (AEE_sd_) in the female as well as the male population. It is noted that adolescents AEE_sd_ significantly decreases with age in both genders (*p* < 0.01).

By use of Spearmans rank correlation coefficient, the interplay between EV, anthropometric indicators of nutritional status (WHR, BMI, and S4SF) and active energy expenditure during school days (AEE_sd_) was assessed.

**Figure 3:**
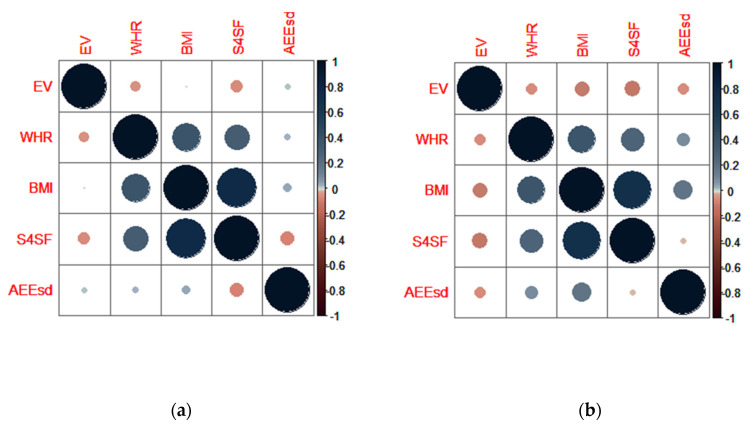
Spearman correlation matrix which shows the interplay between eveningness in energy intake (EV), anthropometric characteristics (WHR, BMI, and S4SF) and active energy expenditure during school days (AEE_sd_) for the male population at the age of: (**a**) 15/16; (**b**) 18/19.

Focusing on the male population, the correlation between EV, anthropometric data, and AEE_sd_ are presented in the correlation matrix separately when participants were 15/16 and 18/19-years old (Figure 3). The interplay between EV and studied variables was not noted at the age of 15/16 (*p* > 0.05). A statistically significant correlation (*p* < 0.01) among anthropometric variables (WHR, BMI, and S4SF) was found. When male participants were 18/19-years old, the interplay between EV and anthropometric variables remained the same, while a statistically significant correlation (*p* < 0.01) was found between AEE_sd_ and BMI values (Figure 3b).

**Figure 4:**
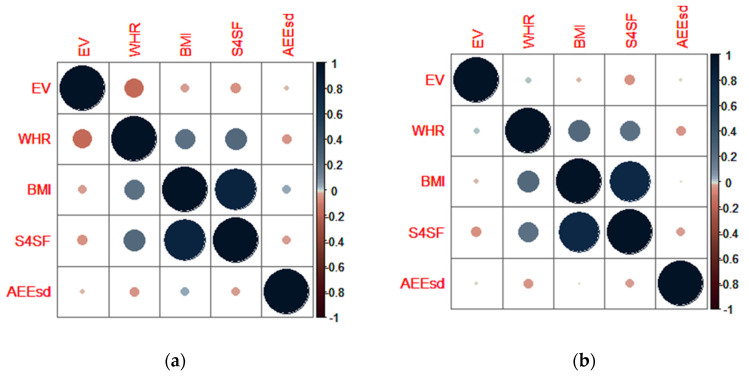
Spearman correlation matrix which shows the interplay between eveningness in energy intake (EV), anthropometric characteristics (WHR, BMI, and S4SF) and active energy expenditure during school days (AEE_sd_) for the female population at the age of: (**a**) 15/16; (**b**) 18/19.

As in males, a statistically significant correlation (*p* < 0.01) among anthropometric variables was noted in the female population—both at the age of 15/16 and 18/19 (Figure 4). The only difference between the female and male population was found in 15/16-years old females showing statistically significant interplay (*p* < 0.01) between EV and WHR (Figure 4a), while the same was not noted when females were 18/19-years old.

The values of statistical significance of correlation (*p*-values) as well as values of Spearmans rank correlation coefficients between variables are available in the Supplementary Data (Tables S1–S8).

**Figure 5:**
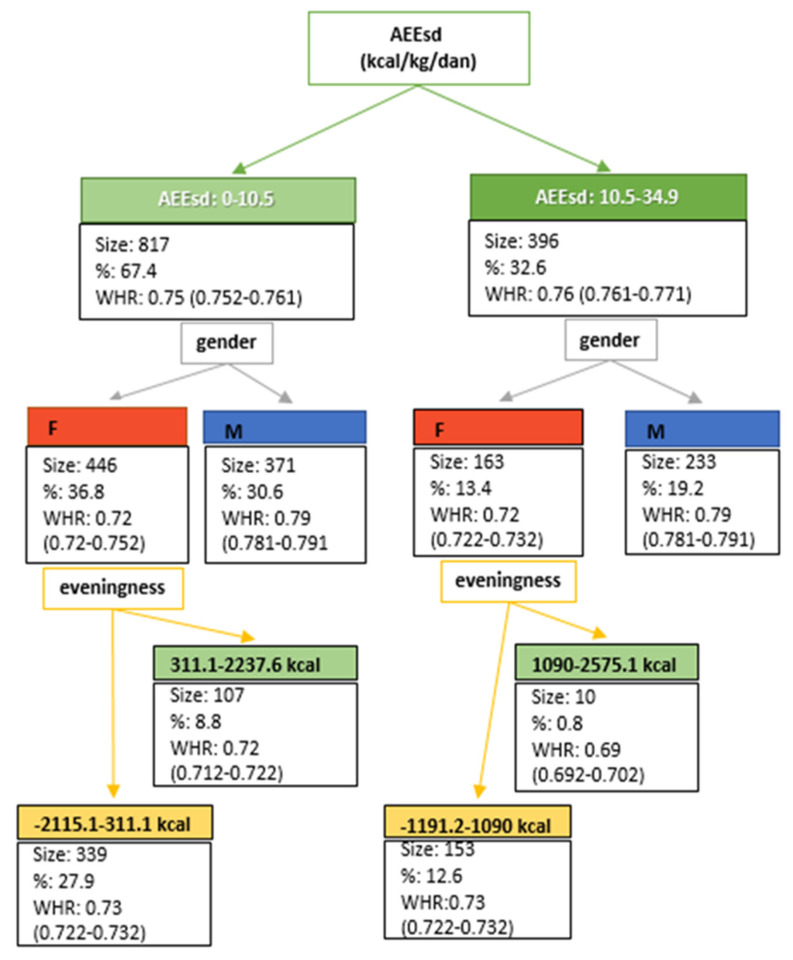
Regression tree, which relates EV to the WHR and AEE_sd_, for students of both genders at the age of 15/16 and 18/19. EV = eveningness in energy intake. WHR = waist to hip ratio. AEE_sd_ = active energy expenditure during school days.

These tables showed an interplay between eveningness (EV), energy expenditure during school days (AEE_sd_), and anthropometric indicators of nutritional status in all age and gender groups (Tables S5–S8). The previously observed significant correlation between WHR and EV among female adolescents at the age of 15/16 prompted us to examine the potential changes in the examined anthropometric size (WHR) if the values of eveningness and physical activity are known. Classification and regression trees are extremely transparent, and their applicability lies in a conceptual model that presented the outputs (Figure 5).

The data used were longitudinal/panel data, and the dependence of repeated observations for the same subject are therefore handled in the analyses using longitudinal analytic methods, such as Generalized Linear models in SPSS. The tools used were General Linear Models (GLM) and General Estimating Equations (GEE) analysis because mixed effect observations can justify how the correlation of repeated observations within each subject was handled [25]. The socio-economic status (SES) is expected to be an important part in such observations. Therefore, we investigated the interaction between SES, age, gender and total daily energy intake (TDEI), as well as their interaction with the observed anthropometric characteristics. All those parameters were observed with the EV as dependent variable. The results of GEE are presented in the Supplementary Data (Tables S9 and S10).

## 4. Discussion

The current study aimed to present differences in specific day-time energy intake, namely EV [12], among female and male Croatian adolescents, participants of the CRO-PALS study, at the age of 15/16 and 18/19. Additionally, the association between various adolescents’ anthropometric indicators of nutritional status and EV was investigated.

Previous studies have shown that energy intake tends to shift towards the later hours of the day as adolescents grow up [12,27], and such a trend was also observed in this study, comparing the whole sample of adolescents at the age of 15/16 and 18/19 (*p* < 0.01). Due to the scarcity of studies examined this trend according to gender, we tested if this phenomenon is equally presented among female and male adolescents. This trend was confirmed in male adolescents (15/16 vs. 18/19) (*p* < 0.01) but was not found in female adolescents (*p* > 0.05). In fact, female adolescents showed the opposite trend—their EV decreases with age. Morning and evening energy intake were defined as in Diederichs et al. (DONALD Study) [12], which allowed for comparison; in the DONALD study, morning energy intake shifted from 24% TDEI among 15/16-year-old adolescents to 23% TDEI among 17/18-year-old adolescents. The corresponding intakes among adolescents at the age of 15/16 and 18/19-years in our study were 26.2% and 25.9%, respectively. Evening energy intake in the DONALD study also shifted as adolescents aged—from 31% in 15/16-year-old to 33% in 17/18-year-old adolescents. The same trend was seen in our study—a shift from 27.1% when adolescents were 15/16 years old to 29.8% when they were 18/19 years old. However, we found that male adolescents, in contrast to female adolescents, showed a significant shift in morning (26.2% to 24.2% TDEI) energy intake as well as evening energy intake (26.0% to 32.1% TDEI) (Figure 1, Table 3), as they age. The increase in EV was in correlation with the increase in TDEI, which was also observed in the DONALD Study [12]—with the exception of female adolescents, where TDEI decreased with the decrease in EV, but the correlation was also noticeable. Of note, a shift in energy intake occured in a period that is specific for the delay in chronotype as a consequence of changes in circadian rhythm [14]. Even though social obligations and daily tasks may delay bedtime, resulting in higher evening energy intake and a higher chance of breakfast skipping, eating patterns of adolescents appear to follow their internal clock rather than socially determined schedules [14]. Unfortunately, the design of this study did not include chronotype evaluation.

The second aim of the study was to examine whether changes in nutritional anthropometric indicators are affected by more pronounced EV. According to the published data, few studies have investigated the connection between pronounced EV, anthropometric indicators of nutritional status, and health implications [28,29,30]. It seems that eating more food at later times of day can lead to deterioration of anthropometric parameters, particularly BMI, but also to a higher risk of chronic and metabolic diseases, such as obesity, metabolic syndrome, non-alcoholic fatty liver disease and diabetes. However, there is a lack of data for adolescents. The present study is, to the best of our knowledge, the first that addressed the impact of energy intake shift, EV, on anthropometric indicators of nutritional status among late adolescents. As adolescents age, from 15/16 to 18/19 years, BMI and the sum of four skinfolds (S4SF) increased among the whole sample population, while waist to hip ratio (WHR) remained the same (Table 4). This is logical, owing to many physiological changes that occur during puberty—increase in energy requirements, body size, metabolic, and hormonal changes [31]. However, when an analysis of body composition was made separately for females and males, it was observed that S4SF decreased within female adolescents with aging (from 47.2 to 46.0 mm, respectively) (*p* > 0.05), which is in contrast with the results of some previous studies [32,33]. Since female adolescents, unlike male, have plausibly higher diet quality, shown as a higher Diet Quality Index for adolescents (DQI-A), and ideal diet score (IDS) [34], the observed decrease in S4SF values could be the result of better adherence to desirable dietary patterns—lower energy density, but higher nutrient density of the food. That assumption was confirmed in the study conducted by Wong et al. [35], where the diet quality of adolescents, aged 14–18 years, from New Zealand, was associated with measures of body fat—higher New Zealand Diet Quality Index for Adolescents (NZDQI-A) scores were significantly associated with lower body fat percentage. Generally, it is known that the desire to be thinner is more common to female adolescents, whereas male adolescents are more occupied with lean muscularity [36]. Consequently, females in adolescence are more likely to take active steps towards restrictive dieting, fasting, or skipping meals in order to lose weight and achieve an ideal body size. Skipping dinner or not eating after certain afternoon hours, as a common method to prevent body weight gain, may also explain the decreasing trend of EV median in female adolescents as they age (Figure 2, Table 3).

Using Spearman’s rank correlation coefficient, we wanted to identify the interplay between EV, anthropometric indicators of nutritional status (WHR, BMI, and S4SF) and active energy expenditure during school days (AEE_sd_), depending on the age and gender of participants. We assumed that participants with higher EV would have more adverse anthropometric parameters, but, on the contrary, the data-processing that was applied showed that EV was inversely correlated with almost every anthropometric parameter independently of gender and age; the correlation between EV and AEE_sd_ was weakly inverse, as expected (Figure 3; Figure 4). However, the significant interplay between EV, anthropometric data and AEE_sd_ was found only in two cases: in females at the age of 15/16, where EV was significantly negatively correlated with WHR (*p* < 0.01), and in males at the age of 18/19, where AEE_sd_ was significantly positively correlated with BMI (*p* < 0.01). In a study by Sila et al. [37], which was also a part of the CRO-PALS study, it was noted that 15/16-year-old females who skip breakfast have a lower TDEI in contrast with females of the same age who have regular breakfast. We speculate that those females, in combination with lower TDEI, unquestionably show higher EV values, with a prospective dominant shift in energy intake. This could explain why the higher EV observed in female adolescents at the age of 15/16, in our study is inversely correlated with WHR; perhaps measures of obesity are more affected by TDEI than by parameters like daily energy intake distribution. Furthermore, as mentioned before, adolescence is a physiologically very dynamic period characterized by gains in muscle mass, predominantly among male adolescents. Larger muscle mass can result in higher BMI and send inaccurate information about body composition. Because of this, it is advisable not to use BMI as a variable for estimating fatness and obesity among adolescents [23]. Additionally, physical activity during adolescence is associated with greater muscle mass [38]. The mentioned observations could partially explain the positive correlation between AEE_sd_ and BMI in male adolescents at the age of 18/19, in our study.

The significance of the correlation (*p*-value) between WHR and EV ranged from 0.002–0.76, and between WHR and AEE_sd_ from 0.17–0.65 (Tables S1–S8). To investigate the inter-relation of these parameters, WHR, EV and AEE_sd_, the classification tree technique was used [39]. This method, the data-mining tool (classification tree), was used since it allows to describe and predict phenomena expressed on a qualitative and quantitative dataset [40]. Classification and regression trees are used to analyze finances [41], medicinal issues [42,43], and in studies related to food [39,40]. However, to our knowledge, classification trees have not been used to study anthropometric indicators of nutritional status related to physical activity and EV. The first part of the tree confirms the stronger correlation of the WHR with the AEE_sd_ than with the EV (Tables S5–S8). The conceptual model (Figure 5) of the regression tree starts with the AEE_sd_ showing that in the majority of the observed population (67.4%), the WHR is expected to be 0.75 if the AEE_sd_ ranges from 0 to 10.5 kcal/kg/day. However, further branching out shows some gender differences, where the average expected WHR in the population is 0.72 (for female population) or 0.79 (for male population), regardless of the AEE_sd_ level. The most interesting branches show the relation between EV and WHR, but only for female adolescents, where 27.9% of them, with an EV ranging from −2115.1–311.1 kcal, are expected to have an average WHR of 0.73.

The importance of the Supplementary Material is an exceptional support for the results presented in the paper itself. In longitudinal studies, it is important to be aware of the methodology which is used in the data analysis [25]. Table S9 confirms that there was no interaction between SES and TDEI with gender and age. Table S10 shows the increase in significance when SES is observed in interaction with WHR and BMI (*p* < 0.05). The BMI change was previously explained based on the significant development and growth, especially in the male population. This result confirms the necessity of the use of GEE/GLM or similar tools to assure that objectivity in reasoning stands on the right foundations.

The results of our study should be interpreted as having certain limitations. Firstly, because of the observational nature of the study, no conclusions on causality can be made, and residual confounding factors cannot be ruled out. Nutritional data were assessed using a single 24-h recall, a method that highly depends on participants’ memory, and a single day, if not representative, does not accurately describe usual intake, however, this was the method of choice considering the logistic challenges, and the limitation was compensated by a relatively large sample size, since the mean intake of a larger group can be estimated by the method [44]. Energy intake, in comparison to macronutrient and micronutrient intakes, also shows less day-to-day variability [45]. In combination with socially desirable reporting, this can lead to under-reporting, but rarely over-reporting, of energy intake [46,47]. Thus, we distinguish “missing” food, food consumed by the respondent but not listed, from “phantom” food, food that the respondent did not consume but stated the opposite [48]. There is also a “flat-slope” syndrome, which occurs in people whose actual food intake is low and tends to overestimate their intake, or when the actual amount of food consumed is high and the respondent tends to underestimate their intake [49]. Moreover, 24-h recalls were collected only for weekdays, excluding Friday, so they do not represent the complete dietary patterns of adolescents and might impact interpretations of energy timing as well as an investigation between energy distribution and anthropometry [50]; however, this was done on purpose. Of note, it is possible that adolescents have higher energy intake on weekend days [51]. The lack of school obligations and social activities may cause EV to be even more pronounced on the weekend days. Another limitation worth addressing is an assessment of physical activity, which was self-reported and, therefore, susceptible to subjective over- or underestimation. Overall, the results of this study show that there is no strong connection between anthropometric parameters and EV as well as AEE_sd_, but some new areas have opened up that certainly need further exploration, perhaps further segmentation of EV with additional cut-offs. However, we did not discuss the absolute values of energy intake and distribution and, instead, we were focused on the relative changes and trends that appeared as adolescents grow. The result of the SES in anthropometric characteristics opens a new topic which will be further examined in following studies. The strengths of our study are reflected in extensive, daytime-specific nutritional data among a relatively large cohort of female and male adolescents as well as in detailed age- and gender-specific anthropometric data. To the best of our knowledge, this study is the first to present an association between EV and several anthropometric nutritional indicators in adolescents as they age, taking into consideration physical activity expenditure.

## 5. Conclusions

The results of the CRO-PALS longitudinal study demonstrated a pronounced shift in energy intake towards later times in the day among adolescents as they age. Eveningness in energy intake (EV) was found to be higher in male adolescents and adolescents in general at the age of 18/19. However, EV was found to be lower in female adolescents at the age of 18/19. When females were 15/16-years old, EV was significantly inversely associated with waist to hip ratio (WHR). Since EV seems to have a minor or no impact on anthropometric indicators of nutritional status and obesity development, subsequent studies investigating not only the timing of dietary quantity, but also dietary quality, total daily energy intake, and chronotype evaluation among adolescents, are warranted. Related to the factor of timing, other parameters beside EV deserve to be tested, or modifications to the definition of EV.

## Figures and Tables

**Table 1:** Croatian physical activity in adolescence longitudinal study (CRO-PALS) study subjects according to age and gender.

Age/Gender	Whole Population	Male	Female
		15–16 years	
Age/years	15.6 (14.4, 16.9)	15.6 (15.0, 16.9)	15.6 (14.4, 16.9)
		18–19 years	
Age/years	18.6 (17.2, 19.9)	18.6 (17.2, 19.9)	18.5 (17.2, 19.9)

Participants age is shown as average (minimum, maximum).

**Table 2:** Age and gender-specific BMI cut-off points depending on the nutritional status of adolescents [22].

Nutritional Status	BMI Percentile Cut-Off Points (kgm^−2^)
Underweight	<5th percentile
Normal weight	≥5th percentile <85th percentile
Overweight	≥85th percentile <95th percentile
Obese	≥95th percentile

**Table 3:** Total daily energy intake, energy intake distribution and eveningness in energy intake according to age and gender.

		15–16 Years			18–19 Years	
**Energy Intake/kcal**	**Whole Population**	**Male**	**Female**	**Whole Population**	**Male**	**Female**
Total daily energy intake (TDEI)	1908 (1409; 2616)	2381 (1815; 3083)	1545 (1193; 2038)	1919 (1363; 2740)	2582 (1909; 3248)	1508 (1196; 1980)
Morning energy intake	495 (283; 792)	625 (363; 947)	409 (230; 618)	475 (305; 753)	596 (356; 900)	395 (279; 594)
(% of TDEI)	26.2 (16.5; 37.5)	26.2 (18.0; 37.3)	26.0 (16.1; 37.6)	25.9 (16.1; 35.6)	24.2 (15.6; 33.7)	27.4 (17.2; 37.9)
Evening energy intake	508 (300; 869)	619 (369; 1040)	424 (267; 668)	567 (320; 940)	769 (471; 1177)	435 (242; 645)
(% of TDEI)	27.0 (17.8; 40.7)	26.0 (17.8; 39.4)	28.2 (17.3; 42.4)	30.2 (19.8; 41.4)	32.1 (22.4; 44.0)	28.2 (17.9; 39.2)
Eveningness in energy intake *	9 (−289; 351)	−3 (−319; 381)	46 (−218; 327)	62 (−241; 425)	198 (−207; 592)	0 (−248; 282)

Energy intake values are show as median (25th; 75th percentile) and as % of TDEI. TDEI = total daily energy intake. * Eveningness in energy intake is defined as the difference between evening and morning energy intake (Equation (5)).

**Table 4:** Sample anthropometric and physical activity data depending on age and gender of subjects.

		15–16 Years			18–19 Years	
Anthropometric Data	Whole Population	Male	Female	Whole Population	Male	Female
BMI/kgm^−2^	21.0 (19.2; 23.2)	20.9 (19.2; 23.0)	21.1 (19.2; 23.4)	21.9 (20.1; 23.9)	22.2 (20.4; 24.3)	21.6 (19.6; 23.3)
% overweight	/	5.6 (25.27)	10.5 (24.19)	/	6.9 (26.46)	10.2 (24.20)
% obese	/	6.0 (28.72)	5.2 (26.89)	/	4.0 (29.52)	6.6 (26.69)
WHR	0.75 (0.72; 0.79)	0.78 (0.76; 0.81)	0.72 (0.69; 0.75)	0.75 (0.71; 0.79)	0.78 (0.76; 0.81)	0.71 (0.69; 0.74)
Sum of 4 skinfolds/mm	39.1 (29.5; 54.1)	30.1 (25.6; 40.4)	47.2 (37.9; 59.3)	39.9 (30.5; 51.6)	32.1 (26.7; 41.0)	46.0 (38.6; 59.1)
Physical activity						
AEE_sd_/kcalkg^−1^	8.1 (5.2; 13.1)	9.5 (6.0; 14.1)	7.3 (4.6; 11.8)	7.0 (4.4; 11.2)	8.2 (5.5; 12.3)	5.9 (3.9; 9.6)

Values are shown as median (25th; 75th percentile). The percentage of overweight and obese participants is shown as a percentage (age and gender-specific BMI percentile cut-off points in kgm^−2^) BMI = body mass index. WHR = waist to hip ratio. AEEsd = active energy expenditure during school days.

## Supplementary Materials

The following are available online at https://www.mdpi.com/2072-6643/12/6/1710/s1, Table S1: Spearman’s rank correlation coefficient for male population at the age of 15/16, Table S2: Spearman’s rank correlation coefficient for male population at the age of 18/19, Table S3: Spearman’s rank correlation coefficient for female population at the age of 15/16, Table S4: Spearman’s rank correlation coefficient for female population at the age of 18/19, Table S5: Statistical significance of correlation (*p*-values) between variables in male population at the age of 15/16, Table S6: Statistical significance of correlation (*p*-values) between variables in male population at the age of 18/19, Table S7: Statistical significance of correlation (*p*-values) between variables in female population at the age of 15/16, Table S8: Statistical significance of correlation (*p*-values) between variables in female population at the age of 18/19, Table S9: Result of the GEE method which included socio-economic status (SES), total daily energy intake (TDEI), gender and age as covariates, Table S10: Result of the GEE method which included the relation between covariates (socio-economic status (SES), total daily energy intake (TDEI), gender and age) and anthropometric characteristics (waist to hip ratio (WHR), body mass index (BMI) and sum of 4 skinfolds (S4SF)).
