# Supplementary material for: Eveningness in Energy Intake among Adolescents with Implication on Anthropometric Indicators of Nutritional Status: The CRO-PALS Longitudinal Study
Source: Nutrients. 2020 Jun 7;12(6):1710. doi: 10.3390/nu12061710 (PMC7352272; doi:10.3390/nu12061710)
Supplement: Supplementary file 1 [file nutrients-12-01710-s001.pdf]

SUPPLEMENTARY DATA

Table S1. Spearman's rank correlation coefficient for male population at the age of 15/16.

Variables	EV	WHR	BMI	S4SF	AEE_{sd}
**EV**	1	-0.05	0.004	-0.07	0.02
**WHR**	-0.05	1	0.39	0.31	0.03
**BMI**	0.004	0.39	1	0.77	0.03
**S4SF**	-0.07	0.31	0.77	1	-0.09
**AEE_{sd}**	0.02	0.03	0.03	-0.09	1

EV = eveningness in energy intake. WHR = waist to hip ratio. BMI = body mass index. S4SF = sum of 4 skinfolds. AEE_{sd} = active energy expenditure during school days.

Table S2. Spearman's rank correlation coefficient for male population at the age of 18/19.

Variables	EV	WHR	BMI	S4SF	AEE_{sd}
**EV**	1	-0.06	-0.11	-0.11	-0.07
**WHR**	-0.06	1	0.37	0.27	0.08
**BMI**	-0.11	0.37	1	0.69	0.17
**S4SF**	-0.11	0.27	0.69	1	-0.02
**AEE_{sd}**	-0.07	0.08	0.17	-0.02	1

EV = eveningness in energy intake. WHR = waist to hip ratio. BMI = body mass index. S4SF = sum of 4 skinfolds. AEE_{sd} = active energy expenditure during school days.

Table S3. Spearman's rank correlation coefficient for female population at the age of 15/16.

Variables	EV	WHR	BMI	S4SF	AEE_{sd}
**EV**	1	-0.17	-0.04	-0.05	-0.01
**WHR**	-0.17	1	0.19	0.24	-0.04
**BMI**	-0.04	0.19	1	0.83	0.03
**S4SF**	-0.05	0.24	0.83	1	-0.03
**AEE_{sd}**	-0.01	-0.04	0.03	-0.03	1

EV = eveningness in energy intake. WHR = waist to hip ratio. BMI = body mass index. S4SF = sum of 4 skinfolds. AEE_{sd} = active energy expenditure during school days.

Table S4. Spearman's rank correlation coefficient for female population at the age of 18/19.

Variables	EV	WHR	BMI	S4SF	AEE_{sd}
**EV**	1	0.02	-0.01	-0.05	-0.004
**WHR**	0.02	1	0.23	0.20	-0.04
**BMI**	-0.01	0.23	1	0.79	-0.003
**S4SF**	-0.05	0.20	0.79	1	-0.04
**AEE_{sd}**	-0.004	-0.04	-0.003	-0.04	1

EV = eveningness in energy intake. WHR = waist to hip ratio. BMI = body mass index. S4SF = sum of 4 skinfolds. AEE_{sd} = active energy expenditure during school days.

Table S5. Statistical significance of correlation (p-values) between variables in male population at the age of 15/16.

Variables	EV	WHR	BMI	S4SF	AEE_{sd}
**EV**	/	0.37	0.94	0.23	0.77
**WHR**	0.37	/	3.21×10^{-12}	4.72×10^{-8}	0.65
**BMI**	0.94	3.21×10^{-12}	/	0	0.56
**S4SF**	0.23	4.72×10^{-8}	0	/	0.12
**AEE_{sd}**	0.77	0.65	0.56	0.12	/

EV = eveningness in energy intake. WHR = waist to hip ratio. BMI = body mass index. S4SF = sum of 4 skinfolds. AEE_{sd} = active energy expenditure during school days.

Table S6. Statistical significance of correlation (p-values) between variables in male population at the age of 18/19.

Variables	EV	WHR	BMI	S4SF	AEE_{sd}
**EV**	/	0.29	0.06	0.05	0.25
**WHR**	0.29	/	1.93×10^{-11}	2.09×10^{-6}	0.17
**BMI**	0.06	1.93×10^{-11}	/	0	0.002
**S4SF**	0.05	2.09×10^{-6}	0	/	0.73
**AEE_{sd}**	0.25	0.17	0.002	0.73	/

EV = eveningness in energy intake. WHR = waist to hip ratio. BMI = body mass index. S4SF = sum of 4 skinfolds. AEE_{sd} = active energy expenditure during school days.

Table S7. Statistical significance of correlation (p-values) between variables in female population at the age of 15/16.

Variables	EV	WHR	BMI	S4SF	AEE_{sd}
**EV**	/	0.002	0.53	0.37	0.83
**WHR**	0.002	/	7.02×10^{-4}	3.34×10^{-5}	0.45
**BMI**	0.53	7.02×10^{-4}	/	0	0.54
**S4SF**	0.37	3.34×10^{-5}	0	/	0.55
**AEE_{sd}**	0.83	0.45	0.54	0.55	/

EV = eveningness in energy intake. WHR = waist to hip ratio. BMI = body mass index. S4SF = sum of 4 skinfolds. AEE_{sd} = active energy expenditure during school days.

Table S8. Statistical significance of correlation (p-values) between variables in female population at the age of 18/19.

Variables	EV	WHR	BMI	S4SF	AEE_{sd}
**EV**	/	0.76	0.81	0.36	0.94
**WHR**	0.76	/	4.37×10^{-5}	5.44×10^{-4}	0.46
**BMI**	0.81	4.37×10^{-5}	/	0	0.97
**S4SF**	0.36	5.44×10^{-4}	0	/	0.49
**AEE_{sd}**	0.94	0.46	0.97	0.49	/

EV = eveningness in energy intake. WHR = waist to hip ratio. BMI = body mass index. S4SF = sum of 4 skinfolds. AEEsd = active energy expenditure during school days.

Table S9. Result of the GEE method which included socio-economic status (SES), total daily energy intake (TDEI), gender and age as covariates.

Source	Wald Chi-Square	Significance
Age	0.342	0.559
Gender	2.105	0.147
TDEI	0.071	0.790
SES	1.012	0.312
Age*Gender	1.340	0.247
Age * TDEI	0.425	0.809
Age * SES	1.012	0.315
Gender * TDEI	1.731	0.188
Gender * SES	0.599	0.439
Age * Gender * TDEI	0.849	0.654
Age* Gender * SES	2.069	0.150
Age * TDEI * SES	0.786	0.675
Gender * TDEI * SES	2.047	0.152
Age * Gender * TDEI * SES	1.365	0.505

Dependent Variable: eveningness (EV)

Table S10. Result of the GEE method which included the relation between covariates (socio-economic status (SES), total daily energy intake (TDEI), gender and age) and anthropometric characteristics (waist to hip ratio (WHR), body mass index (BMI) and sum of 4 skinfolds (S4SF)).

Source	Wald Chi-Square	Significance
WHR	4.933	0.026
BMI	4.078	0.043
S4SF	1.315	0.252
SES * WHR	11.681	0.020
SES * BMI	10.637	0.031
SES * S4SF	1.379	0.848
Gender * WHR	0.005	0.944
Gender * BMI	0.834	0.361
Gender * S4SF	0.023	0.880
Age * WHR	0.774	0.379
Age * BMI	1.951	0.162
Age * S4SF	1.092	0.296
TDEI * WHR	0.010	0.921
TDEI * BMI	0.776	0.378
TDEI * S4SF	0.003	0.956
TDEI * SES	7.111	0.213

Dependent Variable: eveningness (EV)
